# Protective effect of melatonin on learning and memory impairment and hippocampal dysfunction in rats induced by high-fructose corn syrup 

**DOI:** 10.22038/IJBMS.2022.65701.14453

**Published:** 2023-01

**Authors:** Arzu Yalcin, Mustafa Saygin, Ozlem Ozmen, Oguzhan Kavrik, Hikmet Orhan

**Affiliations:** 1 Department of Physiology, Faculty of Medicine, Suleyman Demirel University, Isparta, Turkey; 2 Department of Pathology, Faculty of Veterinary Medicine, Burdur Mehmet Akif Ersoy University, Burdur, Turkey; 3 Department of Biostatistics and Medical informatics, Faculty of Medicine, Suleyman Demirel University, Isparta, Turkey

**Keywords:** High fructose corn syrup, Hippocampus, Learning, Memory, Melatonin

## Abstract

**Objective(s):**

We investigated the harmful effects of high fructose corn syrup (HFCS) on learning and memory in the hippocampus and the ameliorative effects of melatonin (Mel).

**Materials and Methods:**

Thirty-six adult male Sprague Dawley rats were divided into three groups: Group I, control; Group II, HFCS; and Group III, HFCS+Mel. HFCS form F55 was prepared as a 20% fructose syrup solution. Rats in HFCS and HFCS+Mel groups were given drinking water for 10 weeks. Rats in the HFCS+Mel group have been given 10 mg/kg/day melatonin orally for the 6 weeks, in addition to HFCS 55. The Morris water maze (MWM) test was applied to all animals for 5 days to determine their learning and memory levels. After decapitation, one-half of the hippocampus samples were collected for western blot analysis, and another half of the tissues were collected for histopathological and immunohistochemical analyses.

**Results:**

In the HFCS group, there was a significant difference between the time to find the platform in the MWM test and time spent in the quadrant between days 1 and 5 (P=0.037 and P=0.001, respectively). In addition, a decreased level of MT1A receptor, TNF-α, iNOS, osteopontin (OPN), and interleukin-6 (IL-6) expressions were significantly increased in the HFCS group. Melatonin treatment reversed MT1A receptor levels and TNF-α, iNOS, OPN, and IL-6 expressions. During the histopathological examination, increased neuronal degenerations were observed in the HFCS group. Melatonin ameliorated these changes.

**Conclusion:**

Consumption of HFCS caused deterioration of learning and memory in adult rats. We suggest that melatonin is effective against learning and memory disorders.

## Introduction

High fructose corn syrup (HFCS) is a sweetener made from cornstarch. Corn sweeteners have several advantages over sugar, such as freshness, no color or flavor, and low price (1-3). Widespread use of the Western diet is believed to play an important role in the development of such diseases as type II diabetes, obesity, hypertension, (4, 5), and Alzheimer’s, as well as in the development of neurodegenerative disorders (6). Several animal and human studies have shown that memory and learning-related disorders in the cognitive area and pathologies in the hippocampus are associated with Western diet consumption (7-11). 

In the last 30 years, the intake of sugars and calorically sweetened drinks has significantly increased (12-14). Detrimental metabolic outcomes of over-consuming calorically sweetened drinks and other sugars and the effects of consuming different added sugars on cognitive function have been shown in different studies (15-17). In particular, the consumption of these diets and the effect of added sugar intake on people’s cognition levels also change. The outcome of using low-fat, complex carbohydrates in the Western diet in human and animal studies is uncertain. (18, 19).

In particular, hippocampal-dependent spatial memory function and neuronal metabolism are adversely affected by two different types of sweeteners: sucrose and high-fructose corn syrup-55 (HFCS 55), which are the most commonly used sweeteners in many countries (20). In these sweeteners, the ratios of fructose to glucose are as follows: sucrose is 50% fructose to 50% glucose; HFCS-55 is 55% fructose to 42% glucose; and glucose and fructose combine to form sucrose, which is linked to HFCS-55. This difference may contribute to the different metabolic effects of the two sweeteners (21). Recent research has shown that in addition to the likelihood of developing metabolic disorders, the intake of a Western diet during adolescence and adulthood further deteriorates learning and memory functions in both animal and human models (22-24). High-fructose corn syrup has been used as a food additive or sweetener in various products we use daily, and its usage has been continually increasing. 

Melatonin (MEL), an M1/M2 receptor analog, is a lipophilic indoleamine hormone mainly synthesized by the pineal gland. It is most consistently found in the suprachiasmatic nucleus (SCN) of the hypothalamus. Melatonin synthesis shows a plasmatic peak during dark hours and plays an important role in maintaining circadian rhythm. (25, 26). Melatonin demonstrates neuroprotective effects along with antioxidant and antiapoptotic properties by its free radical scavenging at the mitochondrial level (27). It is thought that a decrease in melatonin levels with age may play an important role in the development of neurodegenerative disorders such as Alzheimer’s disease (28). Animal studies have shown that exogenous melatonin not only improves learning and memory, but also reduces ROS production, oxidative stress, and apoptosis that develop with age (29, 30). 

This study was designed to investigate the effects of HFCS-55 consumption on cognitive and metabolic outcomes in adults in a rat model. To investigate the potential neurobiological mechanisms of cognitive deficits, we studied the effects of added sugar intake on the protein markers of inflammation in the hippocampus, a brain region that plays a critical role in spatial memory and learning (31). The present study aimed to investigate the effect of high fructose corn syrup on the hippocampus through learning and memory mechanisms by evaluating MT_1A _receptor expressions. 

## Materials and Methods

***Chemicals***

HFCS was obtained from a local company (Toposmanoglu, Isparta, Turkey), which contained about 24% fructose and 28% dextrose in 73% syrup total solids. Melatonin used for the treatment was purchased from Sigma (USA). 

***Experimental conditions***

Animal treatment and experimental procedures were performed in accordance with the guidelines and regulations for animal care and testing of the European Communities Council Directive (86/609 / EEC), and all the procedures were approved by the Animal Experiments Local Ethics Committee of Mehmet Akif Ersoy University (Number 30.11.2016/241).Thirty-six male Sprague Dawley rats (12-16 weeks old) weighing 250–350 g were obtained from the Animal Investigation Laboratory, Kobay (Ankara, Turkey). The rats were separately housed in solid floor cages with wood chips in a temperature-controlled (23 °C ±1 °C) room for 1 week. During training and until each experimental procedure, the rats were given *ad libitum* access to food and water, with food quantity enough to maintain the rats’ body weight. On the days of the experiment, food and water were provided after the testing. One week prior to the start of the experiment, the rats were adapted to the laboratory conditions: 12/12 hr light/dark cycle (lights off at 08:00 a.m.). 

***Experimental design***

In the study, thirty-six rats were randomly divided into three groups of twelve rats. The groups were as follows:

Group I**:** Control - the rats were fed with a standard commercial diet and tap water for 10 weeks.

Group II**:** HFCS was prepared as 20% (w/v) solutions and given to the rats dissolved in drinking water *ad libitum* for 10 weeks. HFCS was composed of 56% fructose, 37% glucose, and 2% higher saccharides approximately as described in reference 32.

Group III**:** HFCS55 (20%) + MEL (10 mg/kg, orally), the rats were given 20% HFCS55 solution for 10 weeks, a daily single dose of MEL, and a single dose/d for the last 6 weeks of the experiment (33). 

At the end of the experiment, the rats underwent the Morris water maze (MWM) test, where their learning and memory abilities were tested. After the test, the animals were sacrificed under anesthesia with 90 mg/kg ketamine (Alfamin, Alfasan IBV) and 10 mg/kg xylazine (Alfazin, Alfasan IBV). Their whole brains were quickly removed, and half of the brain was collected during the necropsy and fixed in a 10% buffered formaldehyde solution for histopathological and immunohistochemical analyses. The hippocampus was carefully removed from the other half of the brain and placed in Eppendorf tubes (Eppendorf AG, Hamburg, Germany) filled with phosphate buffer (50 mmol / l) and frozen at -20 °C until biochemical analysis (MT_1A_ receptor). 

***Morris water maze (MWM) design***

The performance in MWM was tested in a dimly lit soundproof room with different visual indications. The task for the rats in MWM was to find a hidden underwater platform in the water pool. A probe trial was performed in MWM 24 hr after the last training test. The main objectives were to evaluate the total time spent finding the hidden platform (learning) and the target quadrant (memory) in seconds. The water was made opaque by adding non-toxic paint. Each trial was recorded and transmitted by a camera (Sony, Tokyo, Japan) mounted on the ceiling in the center of the pool. Data were examined using a Smart v3.0 tracking system (Panlab, S.L.U., Barcelona, Spain). The maze was divided into 4 equal quadrants, where each quadrant was numbered; the target quadrant was the fourth one. The platform was in a fixed location during the training and experiment, the daily training consisted of 4 trials. Daily sessions were performed for 5 consecutive days. Using the Morris protocol, we completed the location learning and navigation strategy using spatial cues from day 1 to 4. Meanwhile, a probe memory test was performed. From day 1 to day 5, the animals were allowed to swim in the pool for 60 sec. The experiment was terminated when the rats reached the platform. Rats that could not find the hidden platform within 60 sec on the first day were left on the platform for 30 sec. At the end of day 5, each rat had a total of 20 training trials. At the end of the test, the time taken to find the platform, time spent in the target quadrant, probe trial, and visible platform test markers were analyzed (34, 35).

***Western blotting***

The gel electrophoresis step is included in western blot analysis to resolve the issue of cross-reactivity of antibodies. Anti-Mt-1A was purchased from BIOSS (bs-0027R, Beijing, China). All other reagents had the highest purity grade. The hippocampus was weighed (2 animals per preparation) and homogenized in a cold buffer (36). 50 mm Tris-HCl [pH 7.50. 0.15 mol/l NaCl, 1% Triton X-100, 1 mmol/l ethylenediaminetetraacetic acid [EDTA], 1 mmol/l ethylene glycol tetraacetic acid [EGTA], 25 mg/ml aprotinin, 25 mg/ml leupeptin, 4 mmol/L p-nitrophenyl phosphate, 10 mmol/l benzamidine, and 1 mmol/l sodium orthovanadate were used as the stock solution. The supernatant fractions were obtained by centrifuging the homogenates at 10,000 x g for 10 min, and one part was taken for protein determination (37). An equal amount of protein (50 mg protein per lane) for each sample was separated in 7.5% mini-gel sodium dodecyl sulfate-polyacrylamide gel electrophoresis, electrophoretically incubated to immobilon membrane (Immobilon-P, Millipore), and incubated in tris-buffered saline containing polysorbate 20 (Tween 20, Sigma-Aldrich, Co) [TBST; 50 mm tris-HCl (pH 7.5 - 8.8)], 150 mmol/L NaCl and 30% bovine serum albumin (BSA) containing 0.1% polysorbate) for 20 min. The blots were further incubated in 1% BSA with anti-MT1A (1/1000) and anti-β-actin (1/1000) and kept overnight. The stains were subjected to an additional 10-minute wash in TBST and incubated with alkaline phosphatase-conjugated monoclonal anti-rabbit IgG (1:10 000) in 1% BSA for 1 hr at room temperature; 3 additional washes in TBST were then carried out for 10 min. The membrane was incubated in 5 ml of fresh reagent solution (LuminataTM Forte, Western HRP Substrate, USA) until color development. Immunoblots were identified by a computerized image analysis system (LI-COR Model: 3600, USA). SDS-PAGE and western blot analyses were performed on 6 independent hippocampus preparations. For each stain, the ratio of subunit / β-actin for all the samples (groups I, III, and other experimental groups at the same time) was calculated. 

***Histopathological examinations***

During the autopsy, one-half of the hippocampal samples were collected. They were fixed in 10% neutral formalin for microscopic evaluation. The samples were then routinely processed by automated tissue processor equipment (Leica ASP300S, Wetzlar, Germany) and embedded in paraffin. The tissue sections were cut at 5-μm thickness using a rotary microtome (Leica RM2155, Leica Microsystems, Wetzlar, Germany). The slides were stained with Hematoxylin-Eosin (HE) and examined under a light microscope. Histopathological changes were graded in a blinded manner and lesions were scored to evaluate the pathological findings. 

***Immunohistochemistry***

The hippocampus sections were then immunostained with interleukin-6 [Anti-IL6 antibody, (ab9324)], inducible nitric oxide synthase [Anti-iNOS antibody (ab15323)], osteopontin [(anti-OPN antibody (ab8432))], and Tumor necrosis factor [TNF-alpha antibody (ab6671)] by the streptavidin-biotin technique. All primary and secondary antibodies were purchased from Abcam (Cambridge, UK). The sections were incubated with primary antibodies for 60 min, and immunohistochemistry was carried out using a biotinylated secondary antibody and streptavidin-alkaline phosphatase conjugate. All primary antibodies were used in 1/100 dilution. Expose Mouse and Rabbit Specific HRP DAB Detection IHC kit (ab80436) was used as a secondary antibody. The antigens were shown using diaminobenzidine (DAB) as a chromogen. The primary antiserum step was omitted. All examinations were performed in a blinded manner. Five different sections in each sample were examined and analyzed and then scored from (-) to (+++) according to staining intensity; (-): no stain, (+): light; (++): moderate, and (+++): severe (38). Morphometric analyses and microphotography were performed using the Database Manual Cell Sens Life Science Imaging Software System (Olympus Co., Tokyo, Japan). 

***Statistical analysis***

Statistical evaluations were performed using IBM SPSS Statistics, ver. 22.0. The descriptive statistics are presented as mean ± standard deviations (SD). The data were examined for a normal distribution using the Kolmogorov-Smirnov and Shapiro-Wilk tests. Kruskal-Wallis and Mann-Whitney U tests were used as nonparametric tests in which the variance was not homogeneous and the sample size was low. One-way analysis of variance (One-way ANOVA) was performed on the data showing normal distribution. *Post hoc* LSD and Bonferroni tests were performed to compare the differences. Differences were considered significant at *P*<0.05. 

## Results

***MWM results***

*Escape latency*

All the data regarding the escape latency is presented in Table 1. There was no statistical difference among the groups in terms of the time interval (*P*>0.05). When the groups were compared with each other on different days, there was a statistically significant difference between day 1 and day 5 (*P*=0.037), and the time interval increased on the 5th day. 

*Time spent in the quadrant*

The statistical data of the time spent in the quadrant are presented in Table 2. A statistically significant difference was found between the groups (*P*<0.05). Comparing the results of each day of the experiment, there was a significant difference in the time spent in the quadrant between days 1 and 5 (*P*=0.001).

*MWM: Spatial memory*

**Figure 1:**
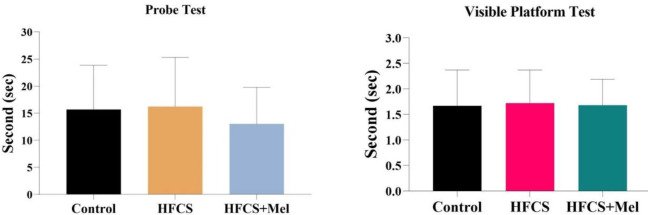
Probe testing and visible platform testing analysis of groups. Values are presented as mean±SD. Relationships between groups and results of platform find time data were assessed by ANOVA test. In probe test and visible platform test was no significant difference was found among Control, HFCS and HFCS groups (*P*>0.05). HFCS: high fructose corn syrup

The memory test was performed as a probe trial after learning blocks. At the end of the HFCS administration, the time spent in the quadrant on the platform (2nd quadrant, target quadrant) and probe trial results were compared between the groups. There was no statistically significant difference between the groups in terms of the time spent in the target quadrant (*P*>0.05) (Figure 1).

*Visible platform test*

In this test, the time (sec) taken to find the platform was evaluated. The visible platform test was performed on day 6 of the learning experiment. Statistical analysis applied to the obtained data showed no significant difference between the groups (*P*>0.05) (Figure 1).

***MT***_1A_ ***receptor expressions in the hippocampus byWestern blot analysis***

**Figure 2:**
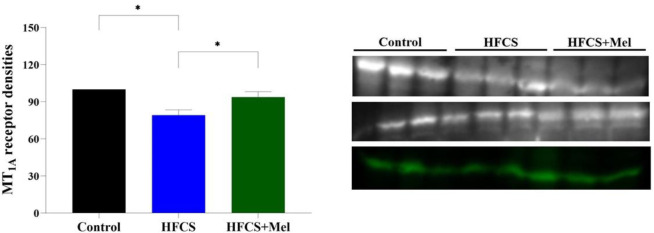
The protein expression levels of MT1A in hippocampus. The relationships between groups were assessed by one-way ANOVA test (*post hoc *Bonferroni test). The MT1A receptor concentration was significantly decreased in the HFCS group compared to the control group (*P<0.001). The MT1A receptor level of the HFCS+Mel group was significantly increased compared to the HFCS group (*P<0.001). *P< 0.001, versus control, HFCS, HFCS + MEL

HFCS: high fructose corn syrup; MEL: melatonin

Melatonergic MT_1A_ receptor expressions in the hippocampal tissue were examined using western blot analysis, and the results were evaluated as the optical density. There was a statistically significant difference between the groups in terms of MT_1A_ receptor levels (*P*<0.001). MT_1A_ receptor level was significantly decreased in the HFCS group compared with the control group (*P*<0.001). The MT_1A_ receptor level of the HFCS+Mel group was significantly increased compared with the HFCS group (*P*<0.001) (Figure 2).

***Histopathological and immunohistochemical results***

*Histopathology findings*

**Figure 3:**
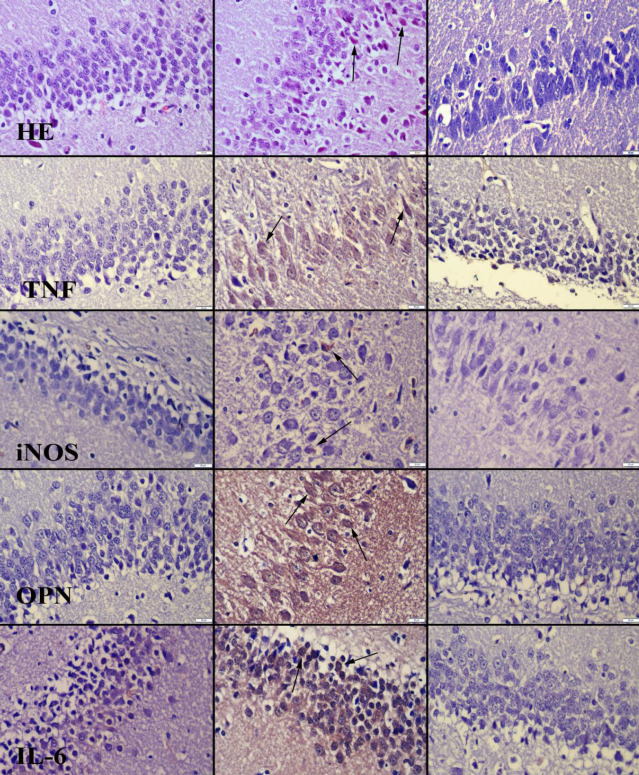
Representative histopathological and immunohistochemical microphotograps between the group. No pathological findings and negative expressions in Control (first colon) group. Increased degenerative neurons and marked expressions in hippocampus in HFCS (second colon) group. Decreased degenerative changes and negative expressions in HFCS+MEL group. HE (first row), streptavidine biotin peroxidase method (second-last rows), Bars=50 µm

HFCS: high fructose corn syrup; MEL: melatonin

There were no pathological findings in the control group (first column). Degenerative neurons in the hippocampus in the HFCS group (second column) and minor degenerative changes in the HFCS+MEL group (third column) were detected histopathologically (Figure 3).

*Immunohistochemical* *findings*

Immunohistochemical evaluation showed increased iNOS, OPN, TNF-α, and IL-6 expressions in hippocampal cells in the HFCS group. However, melatonin ameliorated immunohistochemical findings (Figure 3). 

## Discussion

The most remarkable findings in our study were based on the data we obtained from the learning and memory tests. The findings revealed that long-term consumption of HFCS reduces learning abilities and causes memory deterioration. MT_1A_ receptor expression in the hippocampus was decreased in the HFCS group and increased in the HFCS+Mel group. In light of these results, it was proved that HFCS deteriorates learning and memory functions. Histopathological and immunohistochemical findings supported these results. HFCS caused pathological changes in the hippocampus, but melatonin ameliorated the pathological findings.

Recently, various neurobiological mechanisms, such as neuroinflammation, decreased neurogenesis, and hippocampal and synaptic plasticity dysfunctions have been attributed to increased adolescent HFCS consumption (39. Hsu *et al*. (2015), performing the Barnes maze test following HFCS administration in adolescent rats, found that hippocampal-dependent spatial learning and memory were negatively affected. The researchers showed that learning and memory impairment is unlikely based on nonspecific behavioral effects, as adolescent HFCS 55 consumption did not impact anxiety in the zero maze or performance in a non-spatial response learning task using the same mildly aversive stimuli as the Barne’s maze (40). Ross *et al*. (2009), in the study on adult male Sprague Dawley rats, suggested that a high fructose diet would impair hippocampal-dependent memory. Fructose impaired retention performance in a spatial water maze probe test (41). Another study showed that the effect of a high-sugar diet on spatial memory and neural plasticity was a result of reduced catecholaminergic stimulation in the hippocampus (42). Recent experimental animal studies have shown that high fructose consumption leads to a reduction in hippocampal neurogenesis and changes in mitochondrial activity, suggesting a potential mechanistic basis for fructose-induced cognitive deficits (43). In our study, no significant difference was found in the escape and swimming speed of rats between different groups in the visible platform trial. However, MT1A receptor levels were significantly decreased in the HFCS group compared with the control group. 

We based our previous studies on the effects of melatonin on hippocampal damage and learning and memory function. In this context, we thought that we could improve impaired learning and memory associated with hippocampal damage caused by HFCS. A study showed that melatonin may control the hippocampal glutamatergic system in a complex manner, which may be regulated by the circadian rhythm, thereby influencing memory formation in the hippocampus (44). In addition, it has been suggested that melatonin’s ability to improve cognitive functions is attributed to its antioxidant action (45). 

In a recent study, researchers showed that melatonin improved spatial learning and memory impairment after isoflurane exposure, and these beneficial effects were associated with a reduction in neuroapoptosis and neuroinflammation after isoflurane exposure (46). Another study demonstrated that melatonin administration can regulate neuronal disorders in mice, possibly through modulation of the N-methyl-D-aspartate (NMDA) receptor (47). One more recent study revealed that melatonin may protect against hippocampal damage and memory function deficit by down-regulating calcium-activated potassium channels in cerebral hypoperfusion rats (48). In our study, exogenous melatonin administration significantly improved the time taken to find the platform and the time spent in the quadrant. In addition, the effects of HFCS on learning and memory were investigated by examining hippocampal MT_1A_ receptor levels. It was determined that the MT_1A_ receptor level in the HFCS+Mel group was significantly increased compared with the HFCS group. MT_1A_ receptor level was significantly decreased in the HFCS group compared with the control group. What we learned from our study results is that melatonin can ameliorate HFSC-induced hippocampal-dependent learning and memory impairment in adult rats.

The hippocampus and adjacent regions are very important for spatial learning and memory. Neuroinflammatory markers in the hippocampus typically indicate pathology findings ranging from hippocampal dysfunction to Alzheimer’s pathology (49). Researchers showed increases in protein expression of the proinflammatory cytokines IL-6 and IL-1β in the dorsal hippocampus, which are important for spatial learning and memory in adolescents exposed to HFCS-55 (50). Hsu *et al*. showed increased levels of IL-1β and IL-6 in the dorsal hippocampus in their study. They studied dietary effects on neuroinflammation and stated that adolescence is a critical developmental period for hippocampal-dependent memory impairment induced by HFCS 55 consumption in rats (40). The current study presents a potential neurobiological mechanism for hippocampal-dependent memory impairment in rats consuming HFCS-55 in adolescence. In this context, we demonstrated by immunohistochemistry that TNF-α, iNOS, OPN, and IL-6 expressions were increased in the hippocampus in adult rats fed with HFCS. Melatonin administration reduced TNF-α, iNOS, OPN, and IL-6 expressions. In addition, histopathological examination demonstrated neuronal degenerative changes were demonstrated histopathologically in adult rats fed with HFCS. Melatonin administration diminished degenerative changes.

**Table 1:** Escape latency statistical data in rats (mean±SE)

**Groups/Days**	**First day**	**Second day**	**Third day**	**Fourth day**	**Fifth day**
Control	0,97±1,57	1,24±0,87	1,67±1,14	1,20±0,43	1,44±0,48^a^
HFCS	0,83±0,73	0,74±0,74	1,35±0,64	1,40±0,51	1,57±0,46^a^
HFCS +Mel	0,57±0,38	0,84±0,41	0,82±0,40	1,40±0,47	1,42±0,25^a^

Values are presented as mean±SD. Relationships between groups and results of escape latency data were assessed by independent t test by ANOVA test. a*P*<0.05, compared to first days

**Table 2:** Quadrant spent time statistical data in rats (mean±SE)

**Groups/Days**	**First day**	**Second day**	**Third day**	**Fourth day**	**Fifth day**
Control	41,2±4,09	22,4±4,37	11,5±3,03	5,7±3,17	2,62±0,73^a^
HFCS	39,2±4,01	23,9±4,02	8,16±2,73	3,17±1,02	2,45±0,86^a^
HFCS +Mel	43,9±4,4	22,8±4,7	8,6±3,47	5,2±1,60	0,89±0,24^ab^

Values are presented as mean±SD. Relationships between groups and results of platform find time data were assessed by ANOVA test. a*P*<0.05, compared to first days. b*P*<0.05, compared to HFCS group (between first days and fifth days)

HFCS: high fructose corn syrup

## Conclusion

We investigated the effects of high-fructose corn syrup on learning and memory via MT_1A_ receptor levels; we also investigated the ameliorative effects of melatonin on HFCS-impaired spatial learning and memory. We conducted the learning test which demonstrated a decrease in Mel MT1A receptor levels; however, increased Mel MT_1A_ receptor levels by melatonin administration proved that melatonin has protective effects against adverse outcomes of HFCS. Exogenous melatonin administration may enhance memory functions by reducing the neurodegenerative changes induced by HFCS in the hippocampus. In line with all the studies, we can make three suggestions: 1: we recommend that adolescents avoid using products containing HFCS for better development and use of long-term memory. 2: We also recommend that governments reduce the quota of high-fructose corn syrup in various foods and add melatonin to foods. 3: Future studies should investigate the pathways of hippocampal damage caused by HFCS.

## Authors’ Contributions

All the authors participated in the design and interpretation of the study, data analysis, and the review of the manuscript. AY and OK conducted the experiment and collected the data; MS and HO were responsible for the data analysis. OO performed histopathological and immunochemical analyses and provided methodological and technical guidance. AY wrote the manuscript, and MS reviewed the manuscript. All authors read and approved the final manuscript. 

## Conflicts of Interest

The authors declare that there are no conflicts of interest regarding the publication of this article.
